# Lectures on the Theory and Practice of Physic

**Published:** 1842-11-12

**Authors:** 


### BIBLIOGRAPHICAL NOTICES.

*Lectures on the Theory and Practice of Physic.* By WILLIAM STOKES, M. D. and JOHN BELL, M. D. Second Edition. Philadelphia, Barrington & Haswell. 2 vols, 8vo. pp. 604, 732.

We notice with much pleasure the appearance of a second edition of this work, which, we are also glad to see, has been amplified to two volumes. The animated, graphic style of Dr. Stokes has given his lectures great popularity in this country. They have been published in several forms, in Journals, Libraries, and now, for the second time, as a distinct work. The additions by Dr. Bell to the present edition are so complete, that it takes in the whole range of subjects usually treated in a "Practice of Medicine." We shall give a more extended notice of the work in our next.

